# Nomenclature of therapies in inflammatory bowel disease: A journey through time and terminology

**DOI:** 10.1093/ibd/izaf334

**Published:** 2026-02-11

**Authors:** Michael Colwill, Kamal Patel, Shaji Sebastian, Shahida Din, Sailish Honap

**Affiliations:** Department of Gastroenterology, St George’s University Hospitals NHS Foundation Trust, London, United Kingdom; Institute of Infection and Immunity, City St George’s, University of London, London, United Kingdom; Department of Gastroenterology, St George’s University Hospitals NHS Foundation Trust, London, United Kingdom; Inflammatory Bowel Disease Unit, Department of Gastroenterology, Hull University Teaching Hospitals NHS Trust, Hull, United Kingdom; Hull York Medical School, University of Hull, Hull, United Kingdom; Edinburgh Inflammatory Bowel Diseases Unit, NHS Lothian, Edinburgh, United Kingdom; Institute of Genetics and Cancer, University of Edinburgh, Edinburgh, United Kingdom; Department of Gastroenterology, St George’s University Hospitals NHS Foundation Trust, London, United Kingdom; Department of Immunobiology, School of Immunology and Microbial Sciences, King’s College London, London, United Kingdom

**Keywords:** monoclonal antibodies, inflammatory bowel disease, ulcerative colitis, Crohn’s disease, international nonproprietary name, INN

## Abstract

The expanding therapeutic landscape of inflammatory bowel disease has highlighted the need for clear and standardized drug nomenclature to support safe prescribing, pharmacovigilance, international communication, and patient understanding. The World Health Organization’s international nonproprietary name system, established in 1953, assigns unique and informative names to medicines. However, the increasing number and diversity of monoclonal antibodies used in inflammatory bowel disease and other diseases have outgrown the capacity of the traditional *-mab* suffix to convey meaningful structural or functional distinctions. In 2021, the international nonproprietary name system was updated to introduce new suffixes, such as *-tug*, -*bart*, *-ment*, and *-mig*, that provide more precise information, although these remain unfamiliar to many clinicians. This narrative review explores how international drug naming conventions have evolved and have been applied within the context of inflammatory bowel disease, from early compounds to contemporary engineered therapies, and examines the rationale and clinical relevance of the updated naming framework. Drawing on historical and current literature, as well as policy documents from the World Health Organization’s international nonproprietary name expert group, this review charts the development and successive reforms of the naming scheme. As inflammatory bowel disease therapies continue to diversify, understanding this evolving nomenclature is increasingly important for safe prescribing and effective communication.

Key Messages *What is already known?*

The International Nonproprietary Names scheme standardizes drug nomenclature to ensure universal recognition and understanding. Historically, stems such as *-mab* were sufficient to convey clinical particulars; however, increasing number and complexity of advanced therapies used in inflammatory bowel disease has necessitated an updated system.

*What is new?*

This article describes the evolution of successive International Nonproprietary Names schemes, their relationship to inflammatory bowel disease therapies, and clarifies the meaning and clinical relevance of the new monoclonal antibody suffixes: *-tug*, *-bart*, *-ment*, *-mig.*

*How this helps patient care?*

International nonproprietary name literacy helps clinicians infer molecular target and mechanism of action from names, supporting safer prescribing, pharmacovigilance, treatment sequencing, and clearer communication with patients.

## Introduction

Standardized nomenclature is a cornerstone of the delivery of modern healthcare. It encompasses diseases, diagnostics, and therapeutics to ensure that clinicians, researchers, and health systems share a single and unambiguous language which is also understood by patients. The World Health Organization’s (WHO) International Nonproprietary Names scheme for medicines is a globally utilized naming system that provides clear and specific rules when naming current and future therapeutics and was first published in 1953.^1^ A universal, standardized naming system is essential for safe prescribing, streamlined clinical trials, regulatory clarity, and robust pharmacovigilance, while also supporting effective patient communication and shared decision making. Patients frequently transition between countries and healthcare systems in which identical pharmacological agents may be marketed under different proprietary names. Reliance on international nonproprietary names (INNs) therefore mitigates the risk of medication errors arising from brand variability; supports accurate therapeutic substitution, particularly between originator products and biosimilars; and facilitates continuity of care across settings. In an era of international research, telemedicine, and expanding artificial intelligence integration, harmonized nomenclature also enables seamless data interoperability and ensures that clinical and scientific information remains reliably interpretable worldwide.^2^

Inflammatory bowel diseases (IBDs), mainly consisting of ulcerative colitis (UC) and Crohn’s disease (CD), are chronic, relapsing and remitting, immune-mediated inflammatory disorders (IMIDs) affecting the gastrointestinal tract. They are characterized by mucosal inflammation and a variable disease course that can significantly impact a patient’s quality of life. While medical treatment options were historically very limited,^3^ in the past two decades the therapeutic landscape in IBD has been completely transformed with the emergence of biologics, biosimilars and novel small molecules.^4^ Multiple agents, with distinct mechanisms of action, are in the later stages of development and are advancing through a rich therapeutic pipeline, indicating sustained expansion of the treatment armamentarium in the years ahead.^5^^,^^6^

The rapid expansion of IBD therapeutics has introduced unprecedented complexity into the chemical structures and mechanisms of action of modern therapies that are employed by physicians to treat not only IBD, but also a wide variety of other conditions including cancer, infections, and other IMIDs. The complexity and proliferation in monoclonal antibodies (mAbs) necessitated an update to the International Nonproprietary Names, adopted in 2021,^7^ in which the once-familiar *-mab* suffix for mAbs was replaced with 4 novel suffixes that confer information regarding both the chemical structure and function of each therapy.

A clear understanding of mAb naming conventions is increasingly important in the day-to-day management of IBD particularly given the importance of mechanistic distinctions when making decisions on optimal treatment choice. Nomenclature provides immediate information on molecular structure, immunogenic potential, and biological target, which are all features that directly influence therapeutic drug monitoring strategies, safety considerations, and the rationale for switching within or between therapeutic classes. Accurate interpretation of drug names enables clinicians to navigate treatment pathways efficiently, avoid mechanistic redundancy, and communicate more effectively with patients and multidisciplinary teams. In this context, nomenclature literacy is not merely academic—it is also a practical tool that supports safe, precise, and personalized IBD care.

Despite this, many healthcare providers remain unfamiliar with the origins and rules that govern drug naming. This narrative review examines the historical development and evolving principles of pharmacological nomenclature as applicable to IBD, highlighting the reasoning behind drug naming conventions and aims to help IBD clinicians interpret the critical information embedded in drug names, which can inform clinical decision making and support pharmacovigilance.

## Early chemical compounds

**Figure 1.**
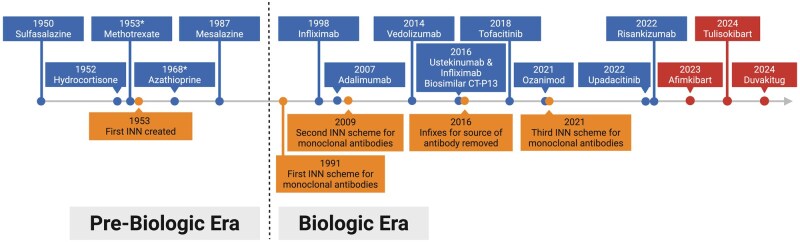
Timeline comparing the dates of U.S. Food and Drug Administration (FDA) approval of selected current and pipeline drugs used for the treatment of inflammatory bowel disease (IBD) alongside milestones in the International Nonproprietary Names (INN) schemes from the World Health Organization. Drugs in red boxes indicate those that have not yet received FDA approval but are being investigated for the treatment of inflammatory bowel disease; the year refers to the first public use of their international nonproprietary name. *Denotes substances not licensed for IBD but widely used “off-label,” and the year refers to FDA approval for other indications. Created in BioRender.

The first drug developed for IBD was sulfasalazine, synthesized in the 1930s by Nanna Svartz (Figure 1),^8^ who initially designed it for rheumatoid arthritis but went on to demonstrate its efficacy in UC.^9^ This represented a major therapeutic breakthrough, transforming symptom control in both conditions.^10^ Its full chemical name—2-hydroxy-5-((E)-2-(4-((pyridin-2-yl)sulfamoyl)phenyl)diazen-1-yl)benzoic acid^11^—is impractical for clinical use, so the simplified name sulfasalazine was created from its components: *sulfa-* for the sulfapyridine moiety, *-sal-* for the salicylic acid responsible for anti-inflammatory activity, and *-azine* for the azo bond linking the two.

The next major therapeutic advance was hydrocortisone, a synthetic form of cortisol first isolated in 1936 and known initially as “compound F.”^12^ It became central to IBD management after the landmark 1955 Truelove and Witts trial in acute severe UC,^13^ a role it retains today.^14^ Its name, like sulfasalazine, predates the International Nonproprietary Names scheme and reflects its chemistry and biological origin: *hydro-* denotes the presence of a hydroxyl group, which enhances membrane permeability and pharmacokinetic properties,^15^ and *-cortisone* identifies it as a synthetic precursor of cortisol, activated hepatically before exerting glucocorticoid and mineralocorticoid effects.^16^ Hydrocortisone remains widely used across multiple conditions, including rheumatoid arthritis, adrenal insufficiency, and severe allergy/anaphylaxis.^17^

Whilst these earlier naming practices were sufficient in the context of a limited number of available therapeutic agents, as drug discovery began to progress at an ever-increasing pace and novel treatments became more widely available, concerns began to arise with regards to heterogeneity amongst products.^18^ The lack of standardization meant that the same drug could appear under many different brand names and a single brand name could even contain different active ingredients.^19^ This created concern with regards to medication errors and potential unwanted drug interactions and it soon became clear that a new and universally utilized system was required.

## The first international naming scheme

These concerns resulted in the establishment of the International Nonproprietary Names program by the WHO, which was first published in 1953.^20^ The primary objective was to standardize pharmaceutical names by assigning each drug a unique, universal designation to promote specificity, clarity, and consistency. Each INN is assigned to the active ingredient itself, not the final commercial product, and is placed in the public domain. This guarantees that the name can be used freely and consistently for clear identification of pharmaceutical substances across countries, languages, and manufacturers. The International Nonproprietary Names system continues to be regularly updated by the INN Expert Group, which meets on a biannual basis.^21^

A key feature of the International Nonproprietary Names system is that it communicates pharmacological relationships through a standardized “stem” approach, which embeds information about a drug’s pharmacological class or mechanism of action directly in its name. This system uses suffixes, infixes, and prefixes as structured “clues” for healthcare professionals to quickly recognize related substances and anticipate potential interactions.^22^

The most important element is the suffix, or main stem, which is mandatory and usually denotes the therapeutic class. Examples include -*pril* for angiotensin-converting enzyme inhibitors (eg, lisinopril), *-olol* for beta-blockers (eg, propranolol), and *-vir* for antivirals (eg, aciclovir). The infix—an affix inserted into the middle of a word that modifies the meaning of the root word—can be inserted before the main stem to provide more specific information and help to refine the classification without changing the core stem. They can convey information such as the drug target, source, or subtype such as *-ast-* representing antiallergic or anti-inflammatory agents such as ebastine, an antihistamine that has been proposed for the treatment of irritable bowel syndrome.^22^^,^^23^ A unique, arbitrary prefix is also added at the beginning of the name to ensure that each INN remains distinct and avoids confusion with existing drug names. The prefix does not carry therapeutic meaning but should ensure that the name will be unique and linguistically acceptable across all languages.

The process of generating an INN was also established at the time of founding the International Nonproprietary Names scheme and continues to this day. Pharmaceutical companies submit several potential names to the INN Expert Group, along with data on the pharmacological particulars of a drug, and the panel then either select one from these suggestions or proposes an alternative.^24^ In contrast, brand naming is subject to far fewer restrictions or processes which allows pharmaceutical companies more creative freedom to produce a brand name for their product. Companies typically aim to produce a unique, memorable and pronounceable name that evokes positive associations or suggests the drug’s function. The names are also designed and tested to ensure they are not associated with negative connotations, potential confusion with other drugs, and prohibited promotional claims.^25^

## IBD treatments and the first naming scheme

Thiopurines were among the earliest IBD therapeutics named under the original INN scheme and exemplify the stem-based structure it introduced. The class name reflects their chemistry where *thio-* denotes a sulfur atom while *-purine* refers to the nitrogen-containing heterocyclic ring found in DNA and RNA. This sulfur-modified purine backbone underpins their role as antimetabolites that disrupt DNA synthesis in rapidly dividing immune cells.^26^ Mercaptopurine follows this convention, with *mercapto-* indicating a sulfydryl-containing mercaptan group and *-purine* identifying its class.^22^ Azathioprine, developed in 1957 and still widely used in IBD, is a prodrug converted to 6-mercaptopurine in which *6*- denotes the position of the thio group on the purine ring.^27^ Its name similarly aligns with stem logic in which *aza-* signals an imidazole ring, *-thio-* the sulfur bridge linking the imidazole and purine groups, and *-purine* again indicating the class.

Methotrexate, also widely used across IMIDs, predates the International Nonproprietary Names system. Synthesized in 1947 as a less toxic analogue of amethopterin, its name primarily reflects chemical structure: *meth-* denotes the methyl substitution reducing toxicity, while *-trexate* refers to a pterin-based folate analogue.^28^ Over time, the International Nonproprietary Names framework incorporated this suffix, and *-trexate* is now recognized as the stem for folic acid analogues, illustrating how the International Nonproprietary Names scheme evolved to integrate disparate naming traditions into a unified system.^22^

Similarly, mesalazine combines chemical description with a pre–International Nonproprietary Names stem: *me-* marks the amino group at the fifth carbon of the salicylic acid ring, while *-salazine* identifies it as a salicylic acid derivative.^22^

## Emergence of biological therapies and the first mAb naming scheme

While the stem structure of the first International Nonproprietary Names scheme provided an effective standardization of nomenclature, the development of the first mAb, muromonab-CD3, in the late 1980s,^29^ and the subsequent rapid expansion in both the number and complexity of these agents, made it clear that an updated naming framework was needed. However, developing such a scheme was challenging because INNs are required to uniquely and simultaneously identify a substance as well as convey information about its activity and these novel mAbs exhibited much greater structural and functional diversity than older therapies. The INN Expert Group therefore agreed on the general suffix *-mab* to indicate mAbs, acknowledging that it would denote shared biochemical structure but not pharmacological action. This system was first applied at the 21st INN Consultation in Geneva in 1991, naming 8 antibodies including biciromab and tuvirumab.^30^ The mAb landscape continued to evolve rapidly, and the biologic era in IBD began with the approval of infliximab in 1998 for use in CD.^31^

Along with the previously described stem structure, infixes were also used to denote the drug’s target, such as *-li-* for the immune system or *-tu-* for a tumor, or the antibody’s source or origin, for example *-xi-* for chimeric (in which the antigen-binding region is nonhuman), *-zu-* for humanized (which combines non–human-derived complementarity-determining regions [CDRs] with human antibody framework regions), or *-u-* for fully human antibodies (entirely derived from human genetic material).^32^ For example, adalimumab is a fully human mAb targeting tumor necrosis factor α (TNF-α) widely used to treated IBD. The -*li*- indicates that it targets the immune system and *-u-* infix reflects its human origin, while in infliximab, another mAb targeting TNF-α and also widely used to treat IBD, *inf-* is the arbitrary prefix and the infixes -*li-* and *-xi-* indicate that it targets the immune system and is chimeric.^19^

## The second mAb naming scheme

By the late 2000s, requests for INNs for mAbs had grown considerably and the system was losing clarity and relevance. The original *-mab* stem, meant for conventional hybridoma-derived antibodies, was increasingly applied to recombinant DNA-produced immunoglobulins, fragments, and engineered variants. Consequently, in 2009 *-mab* was expanded as part of the second INN to cover all substances containing an immunoglobulin variable domain binding a defined target, whether full antibody, fragment, or a novel construct.

While this system offered a degree of clarity, it quickly became unwieldy. As the number of therapeutic mAbs available to treat IMIDs including IBD proliferated, targeting not only TNF-α, but also interleukin (IL)-12/23 (ustekinumab), integrins (vedolizumab), and others, the subtleties in their names often required more expertise to interpret. For example, in ustekinumab, the *-kin*- indicates that it targets the immune system with the *-u-* signifying a fully human source as in adalimumab. Advances in antibody engineering also began to blur molecular distinctions, and the source infix no longer necessarily correlated with manufacturing techniques.^33^ Some manufacturers even allegedly designed antibodies to secure the more marketable -*zu-* or *-u-* infixes that were perceived as superior, even though many effective “chimeric” products (eg, rituximab, infliximab, cetuximab) existed.^7^

To reduce confusion and accommodate the surge of new agents, the source infix was discontinued in 2016 and the antibody’s origin would instead be specified descriptively in the INN definition paragraph.^34^ To avoid conflicts with earlier names, the letters -*u-*, -*o-*, and the syllables -*xi-* and -*zu-* were discouraged, although not prohibited, in direct combination with *-mab*. However, this practice was not always adhered to, and these infixes remained permissible in some cases, such as the drug mirikizumab, an IL-23 p19 inhibitor and a recent addition to the armamentarium in IBD.^35^

## Biosimilars

In the mid-2010s, with the expiry of patents for originator anti-TNF-α biologics and the advent of biosimilars, debates regarding the INN scheme on how to name these new agents also emerged. The U.S. Food and Drug Administration supported the adoption of a naming convention that added a “biosimilar qualifier” suffix to the core names (eg, infliximab-dyyb).^36^^,^^37^ However, critics argued the suffixes were arbitrary and potentially stigmatizing, and this conjecture, combined with the fact that biosimilars are not deemed to have any meaningful therapeutic differences from their bio-originators, meant that this practice was not widely adopted outside the United States.^38^ The European Medicines Agency, for example, advocated for originators and biosimilars to share the same INN.^39^ Consequently, the naming of biosimilars remains largely aligned with that of their originators.

## The third naming scheme

By 2021, the *-mab* stem had become overcrowded, with almost 900 INNs resulting in increasingly long and hard-to-distinguish names and its meaning became inconsistent, encompassing everything from small antibody fragments to complex multispecific constructs. This ambiguity risked medication errors and diminished the clinical utility of the stem.^40^ The WHO INN Expert Group therefore retired *-mab* and introduced 4 new stems to classify antibodies more precisely, which are discussed below (Table 1).

**Table 1.** A simplified summary of the meanings of the new suffixes created by the third International Nonproprietary Names scheme that have replaced the previous *-mab* stem.

Suffix	Meaning	Key features
***-tug***	**Unmodified immunoglobulins**	Single biologic target Full-length antibodies of any class (IgG, IgA, IgM, IgD, IgE) with no major change to their structure Includes most traditional monoclonal antibodies
***-bart***	**Artificial immunoglobulins**	Monospecific and full-length antibodies Undergone molecular engineering with amino acid changes to improve their function (eg, improved stability, efficacy, or pharmacokinetics)
***-ment***	**Immunoglobulin fragments**	Pieces of antibodies that have retaining an antigen binding site May not include the Fc or “tail” section of the structure
***-mig***	**Multispecific immunoglobulins**	Full-length or fragmentary antibodies Designed to bind to 2 or more targets at the same time Different variable domains confer distinct binding sites

Fc refers to the fragment crystallizable region (the stem of the antibody molecule that does not bind to the antigen, but rather interacts with immune cells and other molecules to trigger an immune response).

The third International Nonproprietary Names system also marked an important milestone in international harmonization, as it was adopted for the first time by the U.S. Adopted Names programme.^41^^,^^42^ Although historical differences between the systems were few, most notably mesalazine (INN) vs mesalamine (U.S. Adopted Names), their formal alignment ensures greater universality and clarity. This harmonization is particularly valuable for international clinical trials and regulatory submissions involving novel agents, where consistent nomenclature reduces ambiguity and supports more efficient, streamlined approval processes.

**Figure 2.**
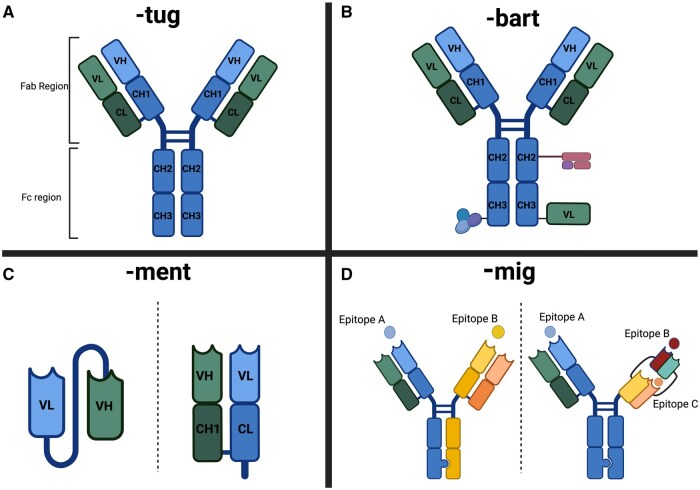
Diagram of the structure of monoclonal antibodies classified by suffix as defined by the third International Nonproprietary Names scheme for monoclonal antibodies: *-tug* for unmodified immunoglobulins; *-bart* for artificial immunoglobulins; *-ment* for immunoglobulin fragments; *-mig* for multispecific immunoglobulins. Created in BioRender. CH, constant region of heavy chain; CL; constant region of light chain; Fab, fragment-antigen binding; Fc, fragment crystallizable; VH, variable region of heavy chain; VL, variable region of light chain.

Applicants requesting an INN for a new biological medicine must now provide the panel with detailed molecular data, including complete amino acid sequences, positions of all disulfide bridges, post-translational modifications, and glycosylation or conjugation profiles.^43^ Novel agents now comprise a unique, invented prefix followed by a standardized suffix denoting pharmacological class, mechanism, or target (Figure 2). The infix, formerly used to indicate the antibody source, is now assigned to reflect mechanism of action or target (eg, *-ki-* denotes a cytokine or cytokine receptor) (Table 2).

**Table 2.** Breakdown of the prefix, infix, and suffix of monoclonal antibodies and small molecules used to treat inflammatory bowel disease.

Drug (International Nonproprietary Names)	Prefix	Infix	Infix meaning	Suffix	Meaning (International Nonproprietary Names elements)
**Original naming scheme**					
**Infliximab**	*inf-*	*-lixi-*	“*li*”—acts on immune system “*xi*”—chimeric	*-mab*	Anti-TNF monoclonal antibody
**Adalimumab**	*ada-*	*-limu-*	“*li*”—acts on immune system “*u*”—fully human	*-mab*	Anti-TNF monoclonal antibody
**Golimumab**	*go-*	*-limu-*	“*li*”—acts on immune system “*u*”—fully human	*-mab*	Anti-TNF monoclonal antibody
**Vedolizumab**	*vedo-*	*-lizu-*	“*li*”—acts on immune system “*zu*”—humanized	*-mab*	Integrin α4β7-monoclonal antibody
**Ustekinumab**	*uste-*	*-kinu-*	“*kin*”—act on IL “*u*”—fully human	*-mab*	Anti-IL-12/23 monoclonal antibody
**Risankizumab**	*risan-*	*-kizu-*	“*ki*”—acts on IL “*zu*”—humanized	*-mab*	Anti-IL-23 p19 monoclonal antibody
**Mirikizumab**	*miri-*	*-kizu-*	“*ki*”—acts on IL “*zu*”—humanized	*-mab*	Anti-IL-23 p19 monoclonal antibody
**Guselkumab**	*gusel-*	*-ku-*	“*k*”*—*acts on IL “*u*”—fully human	*-mab*	Anti-IL-23 p19 monoclonal antibody
**Tofacitinib**	*tofa-*	*—*	—	*-citinib*	JAK inhibitor—“*citinib*” small-molecule kinase inhibitor
**Upadacitinib**	*upa-*	*—*	—	*-citinib*	JAK inhibitor—“*citinib*” small-molecule kinase inhibitor
**Filgotinib**	*fil-*	*—*	“*goti′—*an infix used for JAK inhibitors	*-nib*	JAK inhibitor—filgotinib uses the older “-*nib*” suffix to indicate a small-molecule kinase inhibitor
**Ozanimod**	*ozan-*	*—*	—	*-imod*	S1P receptor modulator—“*imod*” immune modulator
**Etrasimod**	*etras-*	*—*	—	*-imod*	S1P receptor modulator—“*imod*” immune modulator
**Olamkicept**	*olam-*	*-ki-*	“*ki*”*—*acts on cytokine or cytokine receptor	*-cept*	Fc-fusion receptor molecule targeting IL-6
**Third Naming Scheme and IBD Pipeline Examples**					
**Tulisokibart**	*tuliso-*	*-ki-*	“*ki*”—acts on cytokine or cytokine receptor	*-bart*	Full-length monospecific antibodies that have artificial engineered regions
**Afimkibart**	*afim-*	*-ki-*	“*ki*”—acts on cytokine or cytokine receptor	*-bart*	Full-length monospecific antibodies that have artificial engineered regions
**Eltrekibart**	*eltre-*	*-ki-*	“*ki*”—acts on cytokine or cytokine receptor	*-bart*	Full-length monospecific antibodies that have artificial engineered regions
**Duvakitug**	*duva-*	*-ki-*	“*ki*”—acts on cytokine or cytokine receptor	*-tug*	Full-length, unmodified immunoglobulins that closely resemble naturally occurring antibodies

The prefix refers to the initial, manufacturer-selected portion of the name, which must be unique, distinctive, and easily pronounceable in accordance with International Nonproprietary Names guidelines. In the traditional monoclonal antibody naming system, one infix identified the disease or target class, while a second infix specified the antibody source; more generally, infix denotes an element inserted between the prefix and suffix that may convey target or structural information. Suffix (or the common stem) indicates the pharmacological class or mechanism as defined by the International Nonproprietary Names system.

Abbreviations: IBD, inflammatory bowel disease; IL, interleukin; S1, sphingosine-1-phosphate; TNF, tumor necrosis factor.

The IBD therapeutic pipeline is increasingly diverse, featuring novel biological agents such as anti-TL1A inhibitors (eg, tulisokibart), and next-generation mAbs, such as bispecific antibodies.^5^ Although these new agents are likely to be incorporated into the IBD therapeutic armory in the near future, their nomenclature—based on the third INN scheme—may appear unfamiliar to many healthcare providers in IBD.^44^ It is also noteworthy that, because suffixes now reflect therapeutic class, agents directed against the same pathway, such as duvakitug and afimkibart, which both target TL1A, may have markedly different names, which may appear counterintuitive to clinicians.

### *Full-length, unmodified immunoglobulins:* -tug

The suffix *-tug* now covers monospecific full-length antibodies of any class (IgG, IgA, IgM, IgD, IgE) with unmodified constant regions encoded by a single natural allele and identical CDRs that bind to the same epitope.^44^ They may include natural antibodies, chimeric and humanized molecules, and antibodies using identical CDRs to target multiple epitopes.^40^ Duvakitug, an mAb that targets TL1A, is an example of such a therapy that has shown promise for the treatment of UC in early trials,^45^ with recruitment for phase 3 trials due to start in late 2025.^46^

### *Artificial immunoglobulins:* -bart

The suffix *-bart* designates monospecific full-length antibodies with engineered amino acid changes in constant regions—such as alterations to the Fc region or FcRn binding sites, hinge modifications, or the addition of glycan sites, which aims to enhance function, stability, or pharmacokinetic properties such as prolonged half-life and reduced immunogenicity.^44^ It also includes molecules with added variable domains sharing the same CDRs and epitope. Tulisokibart, a novel mAb that targets TL1A and is currently undergoing phase 3 trials in UC^47^ and CD,^48^ and afimkibart, which targets TL1A and is also being investigated in phase 3 trials in UC^49^ and CD,^50^ are both named under this classification.

### *Immunoglobulin fragments:* -ment

The suffix *-ment* describes monospecific antibody fragments not classifiable as *-tug* or *-bart*. These retain at least 1 immunoglobulin variable domain and can have complete, partial, or absent constant regions (eg, Fc-free fragments).^44^ No mAbs from this class are currently licensed for the treatment of IBD.

### *Multispecific immunoglobulins:* -mig

The suffix *-mig* refers to bi- or multispecific antibodies, whether full-length or fragmentary, in which different sets of CDRs confer distinct binding specificities.^44^ It excludes antibodies whose single CDR set happens to cross-react with multiple targets. While no mAbs from this class are currently licensed for the treatment of IBD, several bispecific antibodies are currently in preclinical or early stages of clinical trials and are likely to become an important therapy in the future.^51^^,^^52^

### Practical implications of the third naming scheme

Understanding the revised monoclonal antibody naming conventions has practical implications for clinicians as the therapeutic landscape in IBD becomes increasingly complex. For example, recognizing a *-bart* agent allows clinicians to anticipate modifications in half-life and immunogenicity, which may influence dosing intervals,^53^ and the potential for drug–drug interactions in which Fc or FcRn modifications can lead to inflammatory reactions or increased apoptosis, impacting the efficacy of other therapies.^54^ Similarly, discriminating between *-tug* and *-mig* suffixes can guide therapeutic sequencing and safety monitoring within a mechanistic class. The structural differences signified by these suffixes mark a fundamental shift from single-pathway inhibition to combined therapeutic targeting in the case of *-mig* agents, a strategy that may be particularly advantageous in treatment-refractory disease or those with concomitant IMIDs.^55^ This shift also has implications for safety and monitoring, as multispecific agents denoted by the *-mig* suffix carry theoretical risks not seen with monospecific antibodies.^55^ Although early data for emerging candidates, such as HXN-1003, which targets both IL-23 and TL1A, are reassuring, further study is required to fully characterize their safety and long-term clinical profile.^56^ Furthermore, INN literacy helps to infer the pharmacological specifics of a novel agent being tested in clinical trials, thereby contextualizing efficacy and safety outcomes within an established framework.

### Small molecules

Small molecules, including JAK inhibitors and sphingosine-1-phosphate receptor modulators, have emerged as important therapeutic classes in the treatment of IBD and other IMIDs. The INN stems designated for these stems are -*citinib* for JAK inhibitors—reflecting their role in cytokine signaling inhibition and *-mod* for sphingosine-1-phosphate receptor modulators, which regulate lymphocyte trafficking through receptor modulation. Under the most recent International Nonproprietary Names scheme, these stems have remained unchanged due to the relatively limited number of approved agents within these classes and the homogeneity in their molecular design and biological targets.^1^ However, as drug development in these classes continues, introducing more structurally diverse molecules and novel mechanisms of action, it is anticipated that the INN nomenclature will require further refinement to accommodate this increasing complexity and ensure precise classification.

## Conclusions

In summary, while INN drug names may appear arbitrary, they are in fact deliberate, information-rich constructs designed to convey important knowledge. This system plays an important role in enhancing clarity and safety across drug development, clinical prescribing, regulation, and pharmacovigilance. Despite the growing sophistication of this framework, many healthcare providers remain unfamiliar with how to interpret INNs or apply this knowledge in clinical practice. This represents a missed opportunity, especially in IBD, in which distinguishing between drugs based on target, structure, or mechanism of action is increasingly vital for optimal treatment selection, sequencing, and patient education. As the IBD therapeutic landscape continues to diversify, and with the advent of complex biologics such as bispecific antibodies and immunoglobulin fragments, the ability to decode the pharmacological clues embedded in a drug’s name will become increasingly important. Greater education of IBD physicians to improve knowledge of, and therefore benefit from, the INN scheme is required. The name of a therapy is not just a label—it is a source of insight, and its interpretation should be recognized as an essential component of modern clinical knowledge.
